# Alterations in lung gene expression in streptozotocin-induced diabetic rats

**DOI:** 10.1186/1472-6823-14-5

**Published:** 2014-01-15

**Authors:** Erik van Lunteren, Michelle Moyer, Sarah Spiegler

**Affiliations:** 1 Pulmonary, Critical Care and Sleep Division, Department of Medicine, Louis Stokes Cleveland Department of Veterans Affairs Medical Center, Case Western Reserve University, 10701 East Boulevard, Cleveland, OH 44106, USA

**Keywords:** Type 1 diabetes, Lung, Gene expression

## Abstract

**Background:**

Diabetes profoundly affects gene expression in organs such as heart, skeletal muscle, kidney and liver, with areas of perturbation including carbohydrate and lipid metabolism, oxidative stress, and protein ubiquitination. Type 1 diabetes impairs lung function, but whether gene expression alterations in the lung parallel those of other tissue types is largely unexplored.

**Methods:**

Lung from a rat model of diabetes mellitus induced by streptozotocin was subjected to gene expression microarray analysis.

**Results:**

Glucose levels were 67 and 260 mg/dl (p < 0.001) in control and diabetic rats, respectively. There were 46 genes with at least ± 1.5-fold significantly altered expression (19 increases, 27 decreases). Gene ontology groups with significant over-representation among genes with altered expression included apoptosis, response to stress (p = 0.03), regulation of protein kinase activity (p = 0.04), ion transporter activity (p = 0.01) and collagen (p = 0.01). All genes assigned to the apoptosis and response to stress groups had increased expression whereas all genes assigned to the collagen group had decreased expression. In contrast, the protein kinase activity and ion transporter activity groups had genes with both increased and decreased expression.

**Conclusions:**

Gene expression in the lung is affected by type 1 diabetes in several specific areas, including apoptosis. However, the lung is resistant to changes in gene expression related to lipid and carbohydrate metabolism and oxidative stress that occur in other tissue types such as heart, skeletal muscle and kidney.

## Background

Type 1 diabetes mellitus has widespread adverse effects on many tissues, including heart, kidney, retina, liver, vasculature, peripheral nerve and skeletal muscle. These perturbations contribute importantly to the heightened morbidity and mortality of subjects with diabetes. The lung is also affected by type 1 diabetes, manifested by declines in diffusion capacity, total lung capacity and forced vital capacity [1-5]. This may contribute to the reduced exercise capacity of humans with type 1 diabetes [6] and account for a portion of the increased sensation of dyspnea in diabetics when ventilation or respiratory efforts are increased [7,8]. Nonetheless, severe lung disease is rarely produced by diabetes, suggesting that the lung is more resistant than other organs to this disorder.

Alveolar microangiopathy is postulated to play a prominent role in the genesis of diabetes-induced lung impairment [9]. However, the cellular events leading to lung impairment, and the reasons for the relative sparing of the lung compared with other organs such as the kidney and the eye, are not fully understood. One area with a particular paucity of information is the manner in which diabetes affects expression of genes in the lung. This contrasts with the extensive information gleaned from gene expression array studies of other tissue types, including pancreas [10], kidney [11-13], liver [14], spleen [15], adipose tissue [14], eye [16], corpus cavernosum [17], heart [18-20] and skeletal muscle [14,21-23]. Perturbations of gene expression by diabetes in these organs are large in number and magnitude, and cover many cellular processes such as carbohydrate and lipid metabolism, oxidative stress, and protein ubiquitination. The purposes of the present study were to a) determine the changes in gene expression in the lung due to streptozotocin-induced diabetes, including alterations in expression of genes in common gene ontology groups and b) examine the extent to which affected processes are similar to those reported for other tissue types or are unique to the lung.

## Methods

Studies were performed on twelve male Wistar rats obtained from Charles River Laboratories (Wilmington, MA). All studies were approved by the institutional animal care and use committee of the Department of Veterans Affairs (Veterans Health Administration) and conformed with NIH guidelines for animal care. The streptozotocin-induced model was similar to that of Hida et al. [24]. At an age of eight weeks, seven animals were injected intraperitoneally with streptozotocin 60 mg/kg dissolved in sodium citrate buffer, and five with buffer alone. Four weeks later they were well-anesthetized with a mixture of intraperitoneal ketamine, xylazine and acepromazine following an all-night fast. Blood obtained from the tail was analyzed for glucose using a glucometer (Lifescan, Milpitas, CA). Fasting blood glucose values were 67 ± 4 mg/dl (range 59 to 76) for the normal animals, and 260 ± 13 mg/dl (range 222 to 313) for the diabetic animals (P < 0.001 by unpaired t test). Both lungs from each animal were removed surgically, placed in RNAlater, and stored at -80°C.

Gene expression array studies were performed similar to previous investigations from our laboratory [20,25,26]. Total RNA was extracted using Trizol (GibcoBRL, Rockville, MD), and the RNA pellets were resuspended at 1 μg RNA/μl DEPC-treated water. This was followed by a cleanup protocol with a Qiagen (Valencia, CA) RNeasy Total RNA mini kit. Total RNA was prepared for use on Affymetrix (Santa Clara, CA) microarrays, according to the directions from the manufacturer. Briefly, 8 μg of RNA was used in a reverse transcription reaction (SuperScript II; Life Technologies, Rockville, MD) to generate first strand cDNA. After second strand synthesis, double strand cDNA was used in an *in vitro* transcription reaction to generate biotinylated cRNA. This was purified and fragmented, following which 15 μg of biotin-labeled cRNA was used in a 300 μl hybridization cocktail which included spiked transcript controls. 200 μl of cocktail was loaded onto Affymetrix RAE 230A microarrays (Santa Clara, CA) and hybridized for 16 hr at 45°C with agitation. Standard post-hybridization washes and double-stain protocols used an Affymetrix GeneChip Fluidics Station 400. Arrays were scanned using a Hewlett Packard Gene Array scanner, and analyzed with Affymetrix MAS 5.0 software. The data have been deposited in the NCBI Gene Expression Omnibus (GEO, http://www.ncbi.nlm.nih.gov/geo/) and are accessible through GEO Series accession number GSE15900.

Statistical analysis of the microarray data utilized Bayesian analysis of variance for microarrays (BAM), using BAMarray software (http://www.bamarray.com) [27]. Genes identified by BAM as having significantly changed expression were then further selected based on consistent and appropriate present and absent calls per Affymetrix software. Subsequently signals were averaged for tissue from the non-diabetic and from diabetic animals, and fold changes were calculated based on average values from each group. Analysis focused on genes whose expression changed at least ±1.5 fold in diabetic compared with control lung tissue, unless indicated otherwise. To assign biological meaning to the group of genes with changed expression, the subset of genes which met the above criteria was analyzed with the Gene Ontology (GO) classification system, using DAVID 2.1 software (http://david.abcc.ncifcrf.gov/) [28,29]. Over-representation of genes with altered expression within specific GO categories was determined using the one-tailed Fisher exact probability modified by the addition of a jackknifing procedure, which penalizes the significance of categories with very few genes and favors more robust categories with larger numbers of genes.

Real-time PCR (RT-PCR) was used to confirm changes in gene expression as described previously [20,25,26]. Testing was done using the same lung tissue that had been used for gene expression arrays. An Applied Biosystems ABI 7900HT unit with automation attachment (Foster City, CA) was used for RT-PCR. This unit is capable of collecting spectral data at multiple points during a PCR run. To execute the first step and make archive cDNA, 3 μg of total RNA was reverse transcribed in a 100 μl reaction using Applied Biosystems enzymes and reagents in accordance with the manufacturer’s protocols. RNA samples were accurately quantitated using a Nanodrop Technologies ND-1000 spectrophotometer (Wilmington, DE). Equal amounts of total RNA were reverse transcribed and then used in PCR amplifications. β-Actin had very little variation in expression across the sample set and therefore was chosen as the endogenous control. Since many of the target genes of interest were signaling molecules and likely to be expressed at low levels, we opted for a low dilution factor so as to create an environment more conducive to obtaining reliable results. The cDNA reaction from above was diluted by a factor of 10. For the PCR step, 9 μl of this diluted cDNA was used for each of three replicate 15 μl-reactions carried out in a 384 well plate. Standard PCR conditions were used for the Applied Biosystems assays: 50°C for 2 min, followed by 95°C for 10 min, followed by 40 cycles of 95°C for 15 sec alternating with 60°C for 1 min each. Values for RNA abundance were normalized for each gene with respect to the endogenous control in that sample, mean values for fold changes were calculated for each gene, and statistical testing was performed with the unpaired t-test.

## Results

There were 46 genes in the lung with diabetes-induced alterations in gene expression levels of at least ±1.5-fold (Table 1). For the vast majority of the genes the magnitude of the changed expression was in the range of ±1.5- to ±2-fold, with only 5 genes that had changed expression exceeding ±2-fold and only 1 gene that had changed expression exceeding ±3-fold. For the group as a whole, the number of genes with decreased expression exceeded the number with increased expression, although the only gene with a change exceeding ±3-fold was one with increased expression. A complete list of these 46 genes is provided in Additional file 1: Table S1 [*intended for online publication only*]. Further analysis was performed using the group of all genes with at least ±1.5-fold changed expression.

**Table 1:** Diabetes altered the expression of a relatively small number of genes in the lung

**Fold change threshold**	**Total number of genes**	**Genes with increased expression**	**Genes with decreased expression**
± 1.5	46	19	27
± 2	5	2	3
± 3	1	1	0

Data are depicted for various fold-change thresholds, and are indicated separately for all genes with significantly changed expression, just those with increased expression, and just those with decreased expression. (n = 5, 7 for control and diabetic, respectively).

The 46 genes with altered expression were assigned to gene ontology (GO) terms using over-representation analysis. There were five specific GO terms or groups of closely related GO terms, which ranged in size from three to eight genes each (Table 2). In addition, there were a number of considerably more general GO terms (eg. extracellular region, regulation of biological process, organismal physiological process), which will not be considered further here. Of the five specific GO terms or groups of GO terms, two were characterized by uniformly increased gene expression (apoptosis and response to stress), one by uniformly decreased gene expression (collagen), and two by both increases and decreases in gene expression (protein kinase activity and ion transport). For all five the magnitude of expression changes of the constituent genes did not exceed ±2-fold. Table 3 lists the individual genes that belonged to the three GO terms or groups with uniform directionality of expression changes as well as their respective fold changed expression values.

**Table 2:** Five major specific gene ontology (GO) groupings with statistically significant over-representation among genes with at least 1.5-fold changed expression in diabetic compared with normal lung

	**Number of genes**	**P Value**
**Specific GO term**		
*Terms Related to collagen*		
Collagen	3	0.0024
Fibrillar Collagen	2	0.025
*Terms Related to Ion Transport*		
Ion Transporter Activity	8	0.014
Cation Transporter Activity	7	0.016
*Terms Related to Apoptosis and Cell Death*		
Programmed Cell Death	5	0.025
Death	5	0.026
Cell Death	5	0.026
Apoptosis	5	0.030
*Terms Related to Response to Stress*		
Response to Stress	5	0.033
*Terms Related to Regulation of Kinase Activity*		
Regulation of Protein Kinase Activity	4	0.036
Regulation of Kinase Activity	4	0.038
Positive Regulation of Protein Kinase Activity	3	0.048

P values reflect statistical significance of each GO term being over-represented among genes with altered expression.

(n = 5, 7 for control and diabetic, respectively).

**Table 3:** Specific genes assigned to the gene ontology groups for which the direction of the expression changes were uniform

**Gene symbol**	**Gene name**	**Gene ID**	**Fold change**
*Apoptosis and Cell Death*			
Lcn2	lipocalin 2	170496	1.94
Gadd45b	growth arrest and DNA-damage-inducible 45 beta	299626	1.73
Klf10	Kruppel-like factor 10	81813	1.63
Ebag9	estrogen receptor-binding fragment-associated gene 9	299864	1.59
Prkaa1	protein kinase, AMP-activated, alpha 1 catalytic subunit	65248	1.58
*Response to Stress*			
Ctgf	connective tissue growth factor	64032	1.77
Gadd45b	growth arrest and DNA-damage-inducible 45 beta	299626	1.73
Cd14	CD14 antigen	60350	1.68
Prkaa1	protein kinase, AMP-activated, alpha 1 catalytic subunit	65248	1.58
Lbp	lipopolysaccharide binding protein	29469	1.56
*Collagen*			
Col15a1	procollagen, type XV	298069	-1.88
Col1a1	procollagen, type 1, alpha 1	29393	-1.85
Col3a1	procollagen, type III, alpha 1	84032	-1.70

**Figure 1:**
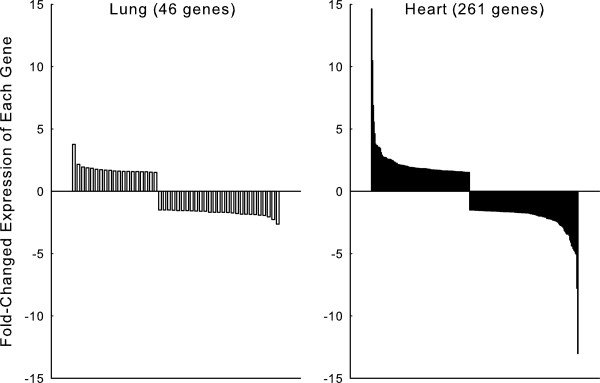
**Gene expression changes with diabetes are compared for the lung (n = 5, 7 for control and diabetic, respectively) and the heart (n = 3, 3 for control and diabetic, respectively).** Plotted are fold-change values for each of the genes with at least ±1.5-fold changed expression in each of the two tissue types. Heart data are from van Lunteren and Moyer (2007).

**Figure 2:**
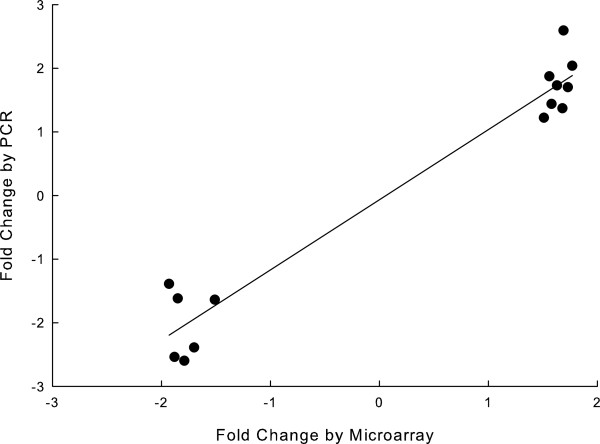
**Changes in gene expression in diabetic lung measured by RT-PCR are compared with values obtained with gene expression microarrays.** All genes had significantly altered expression by microarrays. (n = 5, 7 for control and diabetic, respectively). Data are well correlated (r = 0.97, P < 0.001).

Figure 1 compares the number and magnitude of gene expression changes in the lung with that reported previously for the heart in a study from our laboratory that used the same model of type 1 diabetes and the same methodology as the present study [20]. The heart had many more genes with diabetes-induced altered expression than the lung (261 *vs.* 46 genes, respectively). Furthermore, the maximum magnitudes of the changes in expression were higher in the heart than the lung (range in fold changes of -13.0 to 14.6 *vs.* -2.6 to 3.8, respectively). Consequentially the disease load index (summation of the absolute magnitude of fold changes for all genes with altered expression) [20,30] was considerably higher for the heart than the lung (583.2 *vs.* 81.5, respectively).Results of RT-PCR studies that confirmed gene expression array data are depicted in Figure 2. The direction of the expression changes were identical for expression array and PCR data, and the gene expression changes measured by PCR were statistically significant (range P < 0.05 to P < 0.001). In addition gene array and PCR data were correlated with each other (r = 0.97, P < 0.001).

## Discussion

The present study used a genome-wide expression approach to characterize alterations in lung gene expression by streptozotocin-induced diabetes in rats. Several specific areas were noted with altered gene expression, three of which had uniform directional changes (apoptosis/cell death, response to stress and collagen) and two of which had heterogeneous directional changes (protein kinase activity and ion transport). Nonetheless, the number of genes with altered expression, and the magnitude of the changed expression, were modest compared with findings in most other (albeit not all) tissue types reported previously [13,15-21,31].

Several GO groups of genes with streptozotocin-induced diabetes altered expression found in the present lung study have previously been identified in gene expression array studies of other organ systems. Diabetes-induced changed gene expression related to apoptosis and/or cell death has been found in spleen [15], skeletal muscle [19], and lens [16]. Gene expression changes related to response to stress have been found in skeletal muscle [19] and lens [16]. Collagen gene expression changes have been found in skeletal muscle [21,31], corpus cavernosum [17], and heart [20]. Thus lung and other tissue types share at least some of the effects of type 1 diabetes on gene expression.

A previous study found differences among tissue types (kidney, heart, skeletal muscle and retina) in the effects of diabetes on gene expression [19]. In the present study, two gene groups identified as having changed expression with diabetes in the lung were not identified as having changed expression in a number of gene expression array studies examining other tissues, namely protein kinase activity and ion transport [13,15-21,31]. Conversely, many GO groups of genes with changed expression in other tissues were not found to have changed expression in the lung. One notable area is that of energy production, including lipid and carbohydrate metabolism, which has been identified as having changed expression with type 1 diabetes in skeletal muscle [19,21], heart [18-20], and kidney [19]. Another area is that of oxidative stress, for which gene expression changes have been described with type 1 diabetes in corpus cavernosum [17] and heart [18,20]. One limitation of such direct comparisons of tissue types is the methodological heterogeneity among studies with regards to the specific model of type 1 diabetes studied, the type of gene expression array, and the data analysis and statistical approaches. However, a previous study of the heart from our laboratory [20] used the same methodological strategies as the present study, and identified cardiac changes related to lipid metabolism, oxidoreductase activity and calcium ion binding which were not found in the lung in the present study. This suggests that lung has tissue-specific gene expression responses to type 1 diabetes in addition to the shared responses already mentioned.

Among the five GO terms or groups of GO terms identified as having changed gene expression in the lung with type 1 diabetes, apotosis/cell death is an attractive candidate process that may contribute importantly to the genesis of functional impairment of the lung. Interestingly, insulin inhibits apoptosis [32] and furthermore tight glycemic control accomplished with exogenous insulin or pancreatic transplantation attenuates and/or reverses type 1 diabetes-induced pulmonary function abnormalities [2,33]. Furthermore, osteopontin-deficient mice with streptozotocin-induced diabetes have milder cardiomyopathy and reduced apoptosis compared with wild-type mice with streptozotocin-induced diabetes [34], supporting the involvement of apotosis in diabetes-induced organ dysfunction (albeit that of cardiac muscle). However, genes belonging to the other four GO terms or groups of GO terms, or even single genes not assigned to specific GO terms, may very well also play important roles in the genesis of pulmonary dysfunction with diabetes.

## Conclusions

In summary, this study demonstrates that diabetes mellitus induced by streptozotocin alters gene expression in the lung of Wistar rats. Directionally uniform changes were noted for genes involved in three areas, apoptosis/cell death, response to stress and collagen, whereas directionally heterogeneous changes were noted for genes involved in the regulation of protein kinase activity and ion transport. Equally notable was the absence of changes in some processes such as carbohydrate and lipid metabolism that are implicated in diabetes-induced dysfunction in other tissues.

## Competing interests

The authors declare that they have no competing interests.

## Authors’ contributions

EvL conceived of the study, participated in its design and coordination and drafted the manuscript. SS carried out the initial gene analysis. MM isolated RNA, finished gene analysis and helped with manuscript. All authors read and approved final manuscript.

## Pre-publication history

The pre-publication history for this paper can be accessed here:

http://www.biomedcentral.com/1472-6823/14/5/prepub

## Supplementary Material

Additional file 1: Table S1 Complete list of genes with statistically significant changes of at least ±1.5-fold in diabetic compared with normal lung. (n = 5, 7 for control and diabetic, respectively). Click here for file
