# Supplementary material for: Alterations in lung gene expression in streptozotocin-induced diabetic rats
Source: BMC Endocr Disord. 2014 Jan 15;14:5. doi: 10.1186/1472-6823-14-5 (PMC3945062; doi:10.1186/1472-6823-14-5)
Supplement: Additional file 1: Table S1 — Complete list of genes with statistically significant changes of at least ±1.5-fold in diabetic compared with normal lung. (n = 5, 7 for control and diabetic, respectively). [file 1472-6823-14-5-S1.doc]

SUPPLEMENTAL TABLE 1: Complete list of genes with statistically significant changes of at least ±1.5-fold in diabetic compared with normal lung. (n=5, 7 for control and diabetic, respectively)

Gene Fold

Symbol Gene Title Gene ID Change

Nr1d1 nuclear receptor subfamily 1, group D, member 1 252917 3.75

Nr1d2 nuclear receptor subfamily 1, group D, member 2 259241 2.17

Lcn2 lipocalin 2 170496 1.94

Gpnmb glycoprotein (transmembrane) nmb 113955 1.88

Ccdc23 coiled-coil domain containing 23 362578 1.84

Ctgf connective tissue growth factor 64032 1.77

Gadd45b growth arrest and DNA-damage-inducible 45 beta 299626 1.73

Slc22a3 solute carrier family 22, member 3 29504 1.69

Cd14 CD14 antigen 60350 1.68

Klf10 Kruppel-like factor 10 81813 1.63

Bhlhb3 basic helix-loop-helix domain containing, class B3 117095 1.62

Ebag9 estrogen receptor-binding fragment-associated gene 9 299864 1.59

Xdh xanthine dehydrogenase 497811 1.59

Prkaa1 protein kinase, AMP-activated, alpha 1 catalytic subunit 65248 1.58

P2rx4 purinergic receptor P2X, ligand-gated ion channel 4 29659 1.58

Lbp lipopolysaccharide binding protein 29469 1.56

Pcyox1 prenylcysteine oxidase 1 246302 1.56

Nek6 NIMA (never in mitosis gene a)-related expressed kinase 6 360161 1.53

Atp1b3 ATPase, Na+/K+ transporting, beta 3 polypeptide 25390 1.51

Tas1r2 Taste receptor, type 1, member 2 29408 -1.51

Lrrn3 leucine rich repeat protein 3, neuronal 81514 -1.51

Ckb creatine kinase, brain 24264 -1.51

Cxcl12 chemokine (C-X-C motif) ligand 12 24772 -1.52

Trpc3 transient receptor potential cation channel, subfamily C, member 3 60395 -1.55

Fxyd1 FXYD domain-containing ion transport regulator 1 58971 -1.56

Sfrp2 secreted frizzled-related protein 2 310552 -1.56

Cd79b CD79B antigen 171055 -1.57

Maged2 melanoma antigen, family D, 2 113947 -1.60

Rarres2 retinoic acid receptor responder (tazarotene induced) 2 297073 -1.60

Chek1 checkpoint kinase 1 homolog (S. pombe) 140583 -1.61

Fthfd formyltetrahydrofolate dehydrogenase 64392 -1.68

Rgs7 regulator of G-protein signaling 7 54296 -1.69

Cish cytokine inducible SH2-containing protein 83681 -1.69

Slc7a10 solute carrier family 7 (cationic amino acid transporter,

y+ system), member 10 114518 -1.70

Col3a1 procollagen, type III, alpha 1 84032 -1.70

Pola2 polymerase (DNA directed), alpha 2 85242 -1.74

Scn3b sodium channel, voltage-gated, type III, beta 245956 -1.79

Cxcl14 chemokine (C-X-C motif) ligand 14 306748 -1.83

Tnc Tenascin C 116640 -1.84

Col1a1 procollagen, type 1, alpha 1 29393 -1.85

Col15a1 procollagen, type XV 298069 -1.88

Slc28a3 solute carrier family 28 (sodium-coupled nucleoside

transporter), member 3 140944 -1.93

Ass argininosuccinate synthetase 25698 -1.93

Scd1 stearoyl-Coenzyme A desaturase 1 246074 -2.05

Vapb vesicle-associated membrane protein, associated protein B and C 60431 -2.28

Mx2 myxovirus (influenza virus) resistance 2 286918 -2.64
